# The Growth and Scope of Voluntary Hospitals in London

**Published:** 1897-10-23

**Authors:** 


### THE GROWTH AND SCOPE OF VOLUNTARY HOSPITALS IN LONDON.

(From a Correspondent.)

### XIV.—THE EXTENSION OF GENERAL HOSPITALS.

The establishment of Middlesex Hospital in 1745 was the last effort for nearly eighty years in the direction of expanding General Hospital work. We have shown that during these eighty years the voluntary hospitals of London increased in size, in efficiency, in variety, and in the organisation and development of wise schemes for the patients' good. London became covered with a network of dispensaries, and special provision was during this period established for sufferers from all those diseases still recognised as requiring separate accommodation. It was a time of incessant activity and inquiry in all matters relating to the treatment of the sick poor. The tendency was to criticise and to inaugurate rather than to follow in the old lines, and hard indeed was the task imposed on hospital managers to uphold and improve the work carried on in general hospitals, unaided by popular enthusiasm and heavily impeded by insanitary buildings and obsolete appliances. The Seamen's Hospital, established in 1821, was the direct outcome of funds subscribed in 1818 for the relief of the distressed sailors with whom the London streets were flooded at this period. Founded for the purpose of benefiting a special class only of the community, and carried on afloat for fifty years in the "Dreadnought," it might seem more suitably classed among Special Hospitals. In plan and treatment, however, its work has been always on the lines of a General Hospital, and it now retains nothing of a special character beyond the profession of the patients.

It is perfectly clear that by the year 1820, in which the "West London Infirmary," afterwards the Charing Cross Hospital, was initiated, the General Hospital accommodation of London was quite inadequate for the population. New neighbourhoods had grown up, and old centres become densely congested. Dispensaries planted thick in some localities were (as may be seen by reference to the map printed in The Hospital,

September 25th) wholly absent from others, and it may well be believed that the hopeful view embraced by the first founders of home visitation among the poor, that wounds, operations, and critical forms of disease could be more safely treated in hovels than in hospitals, had given place to a juster conception of the drawbacks to home attendance.

It is curious to note that the moving spirit in the revival of General Hospital work, Dr. Golding, began his work among the poor in St. Martin's Lane and the surrounding district without apparently the smallest conception of the vast organisation of medical relief already possessed by Londoners. This feature of naïveté, often observable in all branches of work among the poor, is specially conspicuous among the promoters of medical charities. Each man, with few exceptions, who has left his mark on voluntary hospital work, has looked no further than his own little circle of sufferers; not co-operation but emulation in good works has been the line pursued, and a feeling of injured surprise instead of delighted recognition is often evoked by the discovery of institutions covering the same ground and sharing the same labours. It cannot be doubted that we have here the gravest defect of the voluntary hospital system in London, yet so inextricably are good and evil intertwined that it is to this narrow burning-glass of enthusiasm, concentrating as it were to one centre the rays of human kindliness and pity, that we owe the institution of most of our noblest charities.

Dr. Golding's work among the poor began as a young man in extensive free practice in his own neighbourhood in order to extend, "by every means in his power, his opportunities of seeing diseases in their multifarious forms and complications." From the year 1815 he turned his house into a kind of free dispensary, to which he surrendered his time daily from eight till one p.m. From the experience thus gained of the needs of the poor, the "West London Infirmary" had its beginning in 1818 as an organisation for home visitation and the free administration of medicine and remedies. From this small beginning the new undertaking rapidly grew in importance. The whole region on either side the Strand was unprovided with dispensaries, and Westminster Hospital, the nearest refuge for accidents, was at an inconvenient distance and in a dilapidated condition. Accordingly, in 1823, a house was taken in Villiers Street to form at first merely the headquarters of the charity; here in 1827 a few beds were fitted up for emergency cases, and the name was changed to the Charing Cross Hospital, and hence in 1834 it was removed to its present site, and commenced its wide sphere of healing and medical instruction. The impulse given to General Hospital expansion did not stop here. Not only did the Westminster Hospital raise large sums simultaneously with the promoter of the Charing Cross Hospital, for reconstructing their institution on its present site, but in the year 1828 the Royal Free Hospital was inaugurated in Greville Street, Hatton Garden. This hospital was the outcome of the increasing demand on the part of the public that the medical relief available for the poor should be absolutely free and fettered by no restrictions. The fees for admission—so much for a clean patient, so much more for a "foul" one, so many pounds for burial in case of death, &c.—imposed by the three endowed hospitals, had been greatly lessened or altogether remitted by the voluntary hospitals; but a letter of admission furnished by a governor of the charity must in all cases be procured, and there were doubtless numerous cases in which eligible patients were deprived of the benefits of the charity for want of friends to procure this passport. The death of a poor unknown girl, aged 18, from disease and want, unaided by any friend, in the winter of 1827, roused the attention of the celebrated surgeon, Mr. William Marsden, to the need for some medical charity touching the lowest stratum of London life; and it was owing to his exertions that the Free Hospital was founded the following year, its great principle being the admittance of all suitable cases without letter or influential recommendation.

The revival of this form of charity is closely linked to the increased activity at this period of medical education and the next general hospital founded, the North London, or University College, owes its origin solely to the demand for clinical material in the medical branch of the university. Its first form was as the "University Dispensary," at No. 4, George street, Euston Square; but this speedily proving insufficient for the needs of the students, a site was procured immediately opposite the university grounds, and in 1833 the first stone of the present building was laid. The hospital was opened the following year. Thus money was forthcoming for the building of four of our best general hospitals simultaneously.

This sustained series of remarkable achievements in the construction of General Hospitals shortly before the opening of this reign concludes with the founding in 1836 of the Metropolitan "Free" Hospital, as it then was, in Bishopsgate. The dates as they stand afford yet another curious illustration of the manner in which public benevolence tends to flow in definite channels moved by springs not always at this distance of time perceptible. During this period from 1820 to 1837 no special hospital was founded, with the single exception of St. Mark's for Fistula in 1835; two dispensaries, the West London Infirmary and the University Dispensary, developed almost immediately on their foundation into general hospitals; two entirely new general hospitals were founded, and older institutions, such as the Westminster and the Middlesex Hospitals, were enabled by the liberality of the public for the first time since their foundation to place the whole of their resources at the disposition of the poor. It was a time of rapid growth and of renewed enthusiasm, and on the foundations thus laid in faith the mighty superstructure of modern science was to be erected.

The following abstract of the nurse's status and privileges at this period will interest many. It is extracted from Erasmus Wilson's "History of Middlesex Hospital," and was the result of an investigation held in the first year of this reign into the conditions of nursing in the principal London hospitals. No diet is given except what is specified :—

St. Bartholomew's.—Sister's average wages, 16s. a week; nurses, 1s. a day, and 12 oz. of bread.

St. Thomas's.—Sisters, £37 a year; nurses, 9s. 7d. a week, and beer.

Guy's.—Sisters, £50 a year; day nurses, £30; night nurses, £20.

St. George's.—Head nurses enter at £21 a year, and have an increase till the wages arrive at £26 5s. The assistants and night nurses enter and continue at £16 a year. All are allowed 6 lbs. of bread per week, ½ pint of milk, and 2 pints of table beer daily, and 1s. a day for board wages.

The London.—Nurses enter at £18 18s., and increase in the women's wards to £23 2s., in the men's wards to £27 6s. a year. Assistant nurses enter at £14 14s., and increase to £17 17s. Gratuities are occasionally paid both to the nurses and the assistants from £1 1s. to £5 5s. a year. The allowance of diet is very similar to that at the Middlesex. [Unfortunately it does not appear what the scale of diet at Middlesex was at this period, but we find the treasurer a few years earlier recommending that on account of the high price of provisions, "the nurses and servants be limited to one half-pound of butter and one pound of cheese each per week," and to reduce the allowance of bread to "one quartern loaf in seven days for each person."]

The Westminster.—Nurses all enter at £16 16s., and may upon special recommendation be increased to £18 18s., but only one such case has occurred in three years. Board entirely, but receive no gratuities.

From the above it will appear that a very varying standard existed in general hospitals relating to the nursing. The result of the enquiry at Middlesex was the expression of a not ill-founded hope "of procuring and retaining (by raising the wages) the better description of nurses—women who will take more interest in their duties, their character, and appearance."

